# Nature-inspired engineering of an artificial ligase enzyme by domain fusion

**DOI:** 10.1093/nar/gkac858

**Published:** 2022-10-16

**Authors:** Cher Ling Tong, Nisha Kanwar, Dana J Morrone, Burckhard Seelig

**Affiliations:** Department of Biochemistry, Molecular Biology and Biophysics, University of Minnesota, Minneapolis, MN 55455, USA; BioTechnology Institute, University of Minnesota, St. Paul, MN 55108, USA; Department of Biochemistry, Molecular Biology and Biophysics, University of Minnesota, Minneapolis, MN 55455, USA; BioTechnology Institute, University of Minnesota, St. Paul, MN 55108, USA; Department of Biochemistry, Molecular Biology and Biophysics, University of Minnesota, Minneapolis, MN 55455, USA; BioTechnology Institute, University of Minnesota, St. Paul, MN 55108, USA; Department of Biochemistry, Molecular Biology and Biophysics, University of Minnesota, Minneapolis, MN 55455, USA; BioTechnology Institute, University of Minnesota, St. Paul, MN 55108, USA

## Abstract

The function of most proteins is accomplished through the interplay of two or more protein domains and fine-tuned by natural evolution. In contrast, artificial enzymes have often been engineered from a single domain scaffold and frequently have lower catalytic activity than natural enzymes. We previously generated an artificial enzyme that catalyzed an RNA ligation by >2 million-fold but was likely limited in its activity by low substrate affinity. Inspired by nature's concept of domain fusion, we fused the artificial enzyme to a series of protein domains known to bind nucleic acids with the goal of improving its catalytic activity. The effect of the fused domains on catalytic activity varied greatly, yielding severalfold increases but also reductions caused by domains that previously enhanced nucleic acid binding in other protein engineering projects. The combination of the two better performing binding domains improved the activity of the parental ligase by more than an order of magnitude. These results demonstrate for the first time that nature's successful evolutionary mechanism of domain fusion can also improve an unevolved primordial-like protein whose structure and function had just been created in the test tube. The generation of multi-domain proteins might therefore be an ancient evolutionary process.

## INTRODUCTION

Naturally evolved proteins often consist of two or more structurally distinct protein domains. These domains are defined by their compact structure and can often fold, function and evolve independently (1,2). Single domain proteins constitute only one-third of all prokaryote proteins and are in the minority in the eukaryote proteome (3). However, natural evolution has used single protein domains as evolutionary modules to produce more complex and sophisticated multi-domain proteins with improved or even new functions through genetic recombination (1,3).

Rational protein design and laboratory directed evolution, have recently been successful in creating entirely artificial enzymes (4–7). In contrast to most naturally evolved proteins, these artificial enzymes are generally single-domain proteins and have lower catalytic activity (6). This initial activity can be improved upon with further directed evolution, yet, these laboratory-based strategies commonly suffer from diminishing returns (8). Often an optimization plateau or local activity optimum is reached, resulting in artificial enzymes that are still inferior to the average naturally evolved enzymes (8,9). An orthogonal evolutionary approach could surmount these activity deficits of artificial enzymes. By emulating natural protein evolution, the function of artificial single-domain proteins could benefit from acquiring suitable auxiliary domains by fusion with other proteins.

DNA and RNA-binding proteins (RNPs) are exemplary illustrations of how natural evolution and protein engineers have perfected enzymes that act on nucleic acid substrates by fusing two or multiple single domain proteins (10,11). For instance, DNA and RNA modifying enzymes consist of catalytic domains that are typically fused to one or more substrate-binding domains, often in a modular arrangement that helps define the fidelity of the binding (10,11). The sequence specificity of many DNA and RNA-binding domains that bind to single stranded or double stranded nucleic acids in a sequence-specific or non-specific manner have been well characterized (11). Examples include zinc-finger domains, helix-turn-helix motifs, RNA recognition motifs and Pumilio domains (12–17). Protein engineers have also fused these binding domains to a variety of proteins to produce improved splicing regulators (18), site-specific endonucleases (19) and translation activators (20,21). However, there are only a few examples where domain fusion has been used to improve overall enzymatic performance without effecting enzyme fidelity. Most notably, the DNA polymerases Taq and Pfu were each fused to the non-specific dsDNA-binding domain Sso7d, increasing polymerase efficiency up to 32-fold without a negative effect on catalytic activity (22). The fusion of the Sso7d domain to Pfu resulted in the commercial Phusion® DNA polymerase. More recently, T4 DNA ligase was fused to various DNA-binding domains, which resulted in a 1.6-fold increase in adaptor ligation efficiency and a 7-fold increase in blunt end cloning. Interestingly, this study also highlighted that different binding domains affect the catalytic efficiency to varying degrees (23). This successful strategy of engineering by domain fusion should be applicable to further enhancing the catalytic activity of artificial enzymes as well.

**Figure 1.**
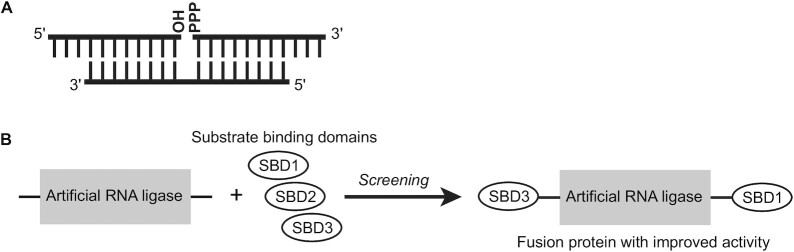
Overview of increasing the catalytic activity of an artificial RNA ligase by fusing the enzyme to terminal substrate-binding domains (SBD). (**A**) The artificial ligase 10C catalyzes a splinted RNA ligation reaction. The 5′-triphosphorylated RNA is ligated to the 3′-hydroxyl of a second substrate in the presence of a complementary DNA or RNA splint. (**B**) A variety of nucleic acid binding domains (4–262 amino acids long) were fused to the N- and/or C-terminus of the artificial RNA ligase 10C (87 amino acids long). The fusion proteins were screened for increased ligation activity.

We previously created an artificial RNA ligase by *in vitro* evolution. Starting from a non-catalytic protein scaffold, a library of 4 × 10^12^ random mutation variants was synthesized and screened for ligase activity using the *in vitro* selection technology mRNA display (6,24,25). This synthetic *de novo* enzyme, ligase 10C, catalyzes the formation of a phosphodiester bond between the 3′-hydroxyl of one RNA and a 5′-triphosphorylated second RNA in the presence of a complementary splint oligonucleotide with the concomitant release of pyrophosphate (6) (Figure 1A). No natural enzyme has been reported to catalyze this reaction. The ligase displays broad sequence specificity and requires zinc (26). A solution structure of the enzyme revealed that the original protein scaffold was lost and that ligase 10C, adopted an entirely new structure (27).

The artificial enzyme accelerates the novel RNA ligation by more than two million-fold over the uncatalyzed reaction. The *in vitro* evolution technique used to generate ligase 10C selected directly for product formation, but not for substrate binding (6,24,25,28). Therefore, suboptimal substrate affinity of ligase 10C likely limited the enzyme's performance and also impeded the full kinetic characterization of the enzyme.

Inspired by the modular design of nucleic acid-binding domains in natural enzymes, we engineered fusion proteins of ligase 10C in order to increase the substrate binding affinity and, consequently, its catalytic activity (Figure 1B). We fused different RNA- and DNA-binding domains and arginine-rich peptides to either, or both, termini of ligase 10C and assayed the ligation activity of the resulting proteins. We then combined the most promising N- and C-terminal domains and performed a detailed characterization of the most catalytically efficient ligase variant.

## MATERIALS AND METHODS

### Generation of gene fusions of ligase 10C and binding domains

The protocol we used to generate each fusion protein differed depending on the size of the domain to be added. For domains that were smaller than 1.5 kDa (rgI, AR_4_, AR_6_), their respective coding sequence was added to the ligase 10C gene during PCR amplification using DNA primers containing the desired sequence. Domains of 1.6–3.7 kDa in length (zk, hzk, tfIII, rgII) were constructed by overlap extension PCR (29). The even longer en domain was fused by amplifying a chemically synthesized gene block from Integrated DNA Technologies, Inc. (IDT) with complementary restriction digest sites and subsequently ligating those amplicons to the ligase 10C gene. Lastly, the rnc domain was amplified from *Escherichia coli* genomic DNA and ligated to the ligase 10C gene. The fusion genes were amplified again by PCR to introduce restriction sites or overlapping sites and cloned into either pET24a or pET28a expression vectors. The primer sequences used to construct each of the fusion domains can be found in Supplementary Table S1. All fusion protein constructs were sequence-verified, and their amino acid sequence is shown in Supplementary Table S2.

### Expression and purification of enzyme variants

The genes for ligase 10C and ligase 10C fusions were expressed in *E. coli* BL21(DE3) cells (Novagen). For the initial activity screening assays, proteins were expressed in 0.5 l of LB medium or 0.2 l of TB medium and purified as described previously (27). Protein concentrations were determined by measuring absorbance at 280 nm using a NanoDrop spectrophotometer 2000c (Thermo Fisher Scientific). Expression of proteins used in experiments to determine *k*_obs_ and *K*_M_ (en-10C-R_4_, 10C-R_4_ and ligase 10C) were carried out at a 6 l scale and at a 2 l scale for the biological replicate. All proteins were stored at 4°C.

### Activity measurements of ligase variants

The α-^32^P-labeled 5′-triphosphorylated RNA (PPP-substrate, 5′-pppGGAACCCAGGUGUUGGUCUUUGACGUAGAGUAUAAGA) was prepared as previously described (27) by *in vitro* T7 transcription of a DNA template consisting of oligonucleotides PPP long forward and PPP long reverse (Supplementary Table S1). The complementary DNA splint #1 (5′-TGGGTTCCGATCGTCG) and RNA-OH #1 substrate (5′-GUUCAGAGUUCUACAGUCCGACGAUC) were purchased from IDT and Dharmacon, respectively. It is important to further purify the gel purified PPP-substrates and the RNA-OH substrates by ethanol-precipitation and an additional 70% ethanol rinse of precipitated substrate pellets to remove potential inhibitors of the ligation reaction.

The single time point assay was performed for the initial screen of the ligation activity of all ligase 10C fusion proteins. Specifically, 10 μM PPP-substrate, 15 μM DNA splint #1 and 20 μM RNA-OH #1 substrate were pre-annealed by heating the mixture at 75°C for 3 min and gradually cooling down to 24°C over a period of 10 min. Each protein (5 μM) was incubated with the pre-annealed substrates at room temperature (21°C) in ligation buffer (20 mM HEPES (pH 7.5), 150 mM NaCl, 100 μM 2-mercaptoethanol and 120 μM ZnCl_2_). After 1 h, the reactions were quenched by mixing them with a solution of 20 mM EDTA and 8 M urea at a 2:1 volume ratio and stored at –20°C. Each sample was thawed and then incubated at 95°C for 5 min and rapidly cooled on ice before loading onto a 20% urea PAGE gel. The ligated product had a lower electrophoretic mobility than the unligated PPP substrate. The formation of product was analyzed using a GE Healthcare Amersham Typhoon Scanner and ImageQuant image analysis software. Statistical differences of the ligation activities of the fusion proteins compared to the unfused ligase 10C were examined by unpaired t-test.

To distinguish between the better-performing fusion enzymes identified in the single time point assay, a multiple time point ligation assay was performed on select fusions enzymes. For this multiple time point assay, we used the same PPP-substrate mentioned above, however with a different RNA-OH substrate, RNA-OH #2 (5′-GUUCAGAGUUCUACAGUCACUAACGUUCGG) purchased from Dharmacon and the matching complementary DNA splint, DNA splint #2 (5′-TGGGTTCCCCGAACGT) purchased from IDT. The substrate concentrations were the same as those used for the initial screen except for an enzyme concentration of 1 μM. Substrates were annealed prior to reaction as described above. Aliquots of the reactions were quenched at indicated time points.

The observed rate constant (*k*_obs_) was determined for the fusion enzymes en-10C-R_4_, 10C-R_4_ and ligase 10C. Product formation was kept below 10% to ensure that only the initial catalytic activity was captured for each enzyme. The reaction conditions and substrate concentrations were the same as in the multiple time point assay described above except for the enzyme concentrations, which was 0.5 μM for en-10C-R_4_ and 10C-R_4_, but 5 μM for ligase 10C. Aliquots of the reactions were quenched at indicated time points. To calculate the *k*_obs_, the slope of percentage ligation over time was multiplied by the ratio of PPP- substrate/enzyme to adjust for the enzyme concentration. It was assumed that all protein was in its active conformation. The reported values are an average of a biological duplicate each with at least three technical replicates as described previously (30).

To measure Michaelis–Menten parameters (*K*_M_ and *k*_cat_) for the most active fusion enzyme en-10C-R4, we determined the *k*_obs_ values from time curves at varying concentrations of PPP-substrate (5–75 μM). For each reaction, the OH-substrate, DNA splint and PPP-substrate were annealed prior to the reaction as described above at a final ratio of 2:1.5:1. The enzyme concentration for all time course assays was kept at 0.5 μM. Reactions were carried out as described above and quenched at 1, 2, 3, 4 and 5 min for the lowest concentration of PPP-substrate (5 μM PPP-substrate) and 1, 2.5, 4, 5.5 and 7 min for all other concentrations of PPP-substrate. The Michaelis-Menten kinetics curve fitting and the kinetics parameters were generated using the GraphPad Prism 6.0 software. It was assumed that all protein was in its active conformation. The reported values are an average of a biological duplicate each with three technical replicates.

### Fluorescence anisotropy assays

Fluorescence anisotropy measurements were conducted at 24°C using a Tecan Spark 10M multifunctional microplate reader. The ligases en-10C-R_4_, 10C-R_4_ and 10C were prepared in ligation buffer (20 mM HEPES (pH 7.5), 150 mM NaCl, 100 μM 2-mercaptoethanol, 120 μM ZnCl_2_). The RNA identical to the ligated product (5′-GUUCAGAGUUCUACAGUCACUAACGUUCGGGGAACCCAGGUGUUGGUCUUUGACGUAGAGUAUAAGA) was prepared as previously described (27) by *in vitro* T7 transcription of a DNA template consisting of oligonucleotides RNA product forward primer and RNA product reverse primer (Supplementary Table S1). The fluorophore-labeled complementary DNA (5′-6-FAM-TGGGTTCCCCGAACGT) was purchased from IDT. We annealed the RNA to the DNA splint in 1:1 molar ratio by heating the RNA/DNA mixture to 95°C and gradually cooling it down to 24°C in an hour. Two-fold dilution series of the enzymes were prepared in ligation buffer also containing bovine serum albumin and the annealed RNA/DNA duplex, resulting in final concentrations of 100 μg/ml and 30 nM for those two components, respectively. All samples had a final volume of 17 μl and were prepared in a low volume 384-well black flat bottom polystyrene microplate (Corning 3821BC). The samples were incubated in the microplate for 90 min. The polarization signals were recorded using excitation and emission wavelengths of 480 and 530 nm, respectively. The instrument G-factor and Z-position for the measurement were calculated from representative wells and the gain was optimized for the measurement. The dilution series was prepared in triplicate. The intensity readings for the wells with only protein sample were subtracted from the intensity readings for each sample well with both protein and the annealed RNA/DNA duplex. The anisotropy value (A) for each sample well was calculated using equation (1). I∥ and I$\bot$ represent parallel and perpendicular fluorescence intensities, respectively, while G represents the relative sensitivities correction factor. The anisotropy data for ligase en-10C-R_4_ (Figure 6C) were fitted to the direct binding model as described in equation (2) (31) using GraphPad Prism 6.0 software. *K*_D_ represents the dissociation constant, L_T_ represents the total concentration of ligand, and P represents the concentration of protein.

(1) $$\begin{equation*}{\rm{A}} = \frac{{{\rm{I}}\parallel - {\rm{G*I}} \bot }}{{{\rm{I}}\parallel - 2{\rm{*G*I}} \bot }}\end{equation*}$$

(2) $$\begin{eqnarray*}\frac{{{\rm{A}} - {{\rm{A}}_{{\rm{min}}}}}}{{{{\rm{A}}_{{\rm{max}}}} - {{\rm{A}}_{{\rm{min}}}}}} &=& {\rm{fraction}}\,{\rm{bound}} \nonumber\\ &=& \frac{{{{\rm{K}}_{\rm{D}}} + {{\rm{L}}_{\rm{T}}} + {\rm{P}} - \sqrt {{{\left( {{{\rm{K}}_{\rm{D}}} + {{\rm{L}}_{\rm{T}}} + {\rm{P}}} \right)}^2} - 4{\rm{*}}{{\rm{L}}_{\rm{T}}}{\rm{*P}}} }}{{2{\rm{*}}{{\rm{L}}_{\rm{T}}}}}\end{eqnarray*}$$

## RESULTS

### Identifying nucleic acid-binding domains to fuse with ligase 10C

Nucleic acid-binding proteins are plentiful in nature (10). We defined a set of criteria to narrow our choices to those domains that we deemed more likely to improve ligation efficiency when fused to ligase 10C. To keep a feasible workload of the project we aimed to test about a dozen domain candidates. Therefore, the search for domain candidates was not exhaustive but rather a sampling of promising candidates. Firstly, we only considered domains that have been described to bind RNA in a non-sequence-specific manner to maintain sequence-independent ligation. This criterion considerably reduced the number of potential nucleic acid-binding domain candidates. Secondly, we focused on binding domains that are smaller than ligase 10C (9.8 kDa). This led to the selection of zinc knuckle (zk), helix zinc knuckle (hzk) and RGG/RG box motifs. Ideally, we wanted to avoid larger domains to prevent a potential occlusion of the catalytic center of ligase 10C. However, many double stranded RNA-binding domains are either of similar or larger size compared to ligase 10C, which led us to select a binding domain from RNase III (rnc) (32). Finally, we also chose an engrailed homeodomain (en) that contains the highly prevalent and evolutionary conserved DNA-binding helix-turn-helix motif (33). All chosen binding domains and their respective acronyms are shown in Table 1.

**Table 1.** Design, expression and ligation activity of protein fusions of ligase 10C with different substrate-binding domains. Expression in *E. coli* and the purification of the soluble fraction by Ni-NTA chromatography was either successful (>90% pure; Y), successful containing impurities (<90% pure; Y*), or yielded no detectable protein (N). Purity was assessed by SDS-PAGE gel electrophoresis (Supplementary Figure S1). Ligase activity of each protein was measured at a single time point (1 hour) by PAGE gel shift assay and was compared to ligase 10C. The symbols +/–, + and ++ indicate ligase activity either similar to ligase 10C at a ligation yield of 14 ± 3%, or 30–40% ligation yield, or more than 40% ligation yield, respectively. Reduced activity was denoted by -, indicating 1–10% ligation yield. The source protein for each domain is also listed. The choice of domains tested and whether they were fused to the N-, C-, or both termini was not comprehensive but a sampling of practical number of constructs

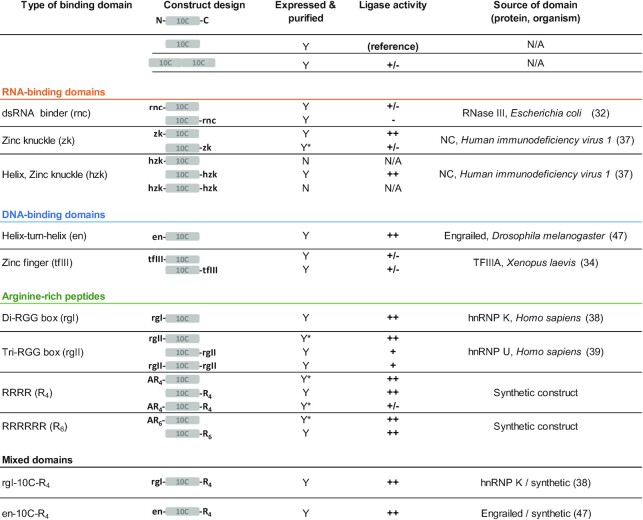

The different types of nucleic acid-binding domains we selected all possessed different binding characteristics. These choices enabled us to compare the performance of different types of binding domains on the catalytic efficiency of ligase 10C. The double-stranded RNA-binding domain contained in rnc from *E. coli* was selected because it can interact specifically with the RNA minor and major groove of the A-form helix adopted by double stranded RNA and an RNA-DNA duplex (32). Zinc fingers motifs constitute one of the largest protein superfamilies. Most zinc fingers have been reported as sequence-specific DNA-binding domains but there are an increasing number of examples showing that zinc fingers also bind to RNA and often in a sequence-independent manner. The TFIIIA protein contains six zinc fingers, the fourth of which we chose to fuse with ligase 10C because it has been shown to bind to RNA in a sequence-independent manner (tfIII) (12,34). Zinc fingers that bind only to RNA can be found in all retroviral nucleocapsid (NC) proteins (35). We chose the well-characterized zinc knuckle domain from the human immunodeficiency virus (HIV) type 1 NC protein, either with the native additional N-terminal helix (hzk), or without (zk) (36,37). The RGG/RG box motif is a more recently identified motif and refers to the high occurrence of amino acid clusters rich in positively charged arginine and glycine that have been observed in many RNA-interacting proteins (38,39). It has been difficult to define RGG/RG box motifs correctly, therefore, as well as selecting known natural RGG/RG box motifs from the hnRNP K and hnRNP U (rgI and rgII) proteins, we also produced synthetic tetra- and hexa-arginine peptide constructs to fuse to ligase 10C (38,39). The synthetic constructs were inspired by the natural RGG motifs and were used as simplified versions of those to test whether varying numbers of positively charged arginine residues alone would be sufficient to increase nucleic acid-binding. The RGG motif represents a very small domain that we hoped would minimize any interference with the enzymatic active site of ligase 10C. The final domain we chose was the engrailed homeodomain (en) that binds to DNA using a helix-turn-helix (HTH) motif. The en is a well-characterized and evolutionary conserved DNA-binding domain that interacts with the DNA major groove in a non-sequence-specific manner (40,41). To the best of our knowledge, an interaction of en with RNA has not been reported. However, our interest in the homeodomain stems from the previously observed striking similarities between the surface binding site of HTH domains and the RNA-binding site on the ribosomal protein, L11 (40,42,43) and the observation of the multifunctional DNA and RNA binding of the bicoid homeodomain protein (44).

### Construction, expression and purification of fusion proteins

We obtained the sequences for the substrate-binding domains from well-characterized nucleic acid modifying proteins with a known crystal structure (Table 1). To produce the fusion proteins, the DNA sequence encoding each domain was fused to either the N- or C-terminus of ligase 10C. To reduce the potential risk of steric interference with the active site of ligase 10C, the domains rnc, zk, hzk, tfIII, en and rgII were fused to ligase 10C via an additional glycine-serine-rich sequence as a flexible linker (5–10 amino acids in length). The amino acid sequence of all fusion constructs is shown in Supplementary Table S2. Using the same expression vectors, *E. coli* growth and purification protocols that had previously yielded soluble ligase 10C, we attempted the production of the ligase 10C fusion proteins (25). The presence of some binding domains clearly affected the expression and solubility of the enzymes. Ligase 10C fusions to either DNA-binding domains or arginine-rich peptides were successfully expressed and purified (Table 1) (Supplementary Figure S1). Expression was less efficient when ligase 10C was fused to the RNA-binding domain rnc (Table 1). Additionally, we found that expression differed depending on the location of the fused domain. For instance, the 10C-rnc fusion expressed well, but there was much lower expression of rnc-10C. The reverse and even more dramatic result was obtained for the hzk domain with 10C-hzk being expressed, but no expression for hzk-10C. Except for the aforementioned examples, all other fusion proteins were soluble and were purified for *in vitro* activity assays. Overall, successful expression and purification appeared to be loosely correlated to the size of the binding domain; larger binding domains having a negative impact on expression, whereas, the addition of smaller domains and small synthetic regions had little to no effect on expression when compared to ligase 10C alone. (Supplementary Figure S1).

### Initial screen of enzymatic activity by single time point assay

**Figure 2.**
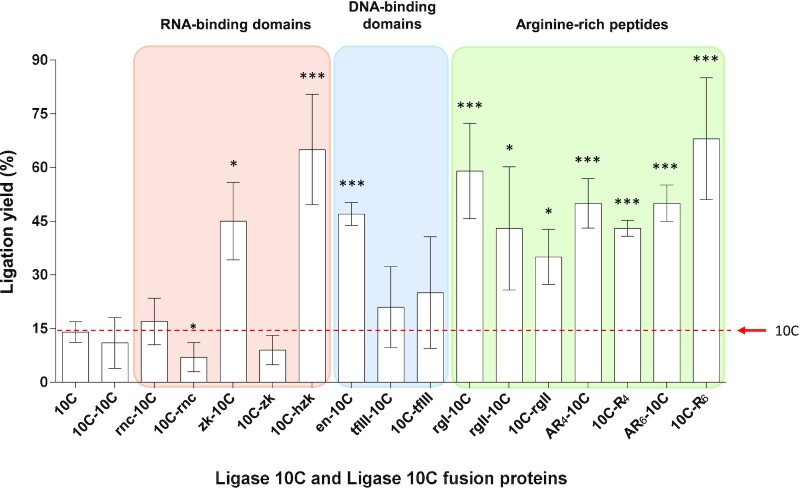
Comparison of ligation activity of ligase 10C with substrate-binding domain fusions of ligase 10C. The fusion proteins are grouped and color-coded according to the type of their binding domain, similar to Table 1. Only fusions with a single substrate-binding domain are shown here. Ligation yields were determined after 1 h incubation. Assays contained 5 μM protein, 10 μM PPP-substrate, 15 μM DNA splint (DNA splint #1), and 20 μM OH-substrate (RNA-OH #1). The error bars represent the standard deviations of the means of four replicates. *P < 0.05 and ***P < 0.001 indicate statistically significant differences of ligation yield when compared to the ligation yield for unfused ligase 10C.

To enable facile screening of the large number of soluble fusion proteins, the ligation activity was determined by a single time point assay. The ligation yield was quantified by a urea PAGE gel shift assay that separated the unligated radiolabeled substrate RNA starting material from the longer ligated RNA product. (Supplementary Figure S2). At the chosen conditions, ligase 10C alone ligated 14 ± 3% of the provided substrate (Figure 2). The ligation yield of ligase 10C was greatly increased when the protein was fused to any of the arginine-rich peptide domains (>35 ± 7.7% yield). The DNA-binding helix-turn-helix domain (en) and the RNA-binding zinc knuckle domain (hzk) also improved the yield to 47 ± 3.1% and 65 ± 15%, respectively, when the en domain was fused to the N-terminus and the hzk domain to the C-terminus of ligase 10C. Interestingly, the RNA-binding zinc knuckle domain (zk), which is very similar to the hzk domain, increased the ligation to 45 ± 11% when fused to the N-terminus and yielded only 9.0 ± 4.1% ligation when fused to the C-terminus. A significant reduction in ligation yield (7 ± 4%) was observed for the RNA binding domain rnc when fused to C-terminus. Finally, no significant change in activity was observed for all the remaining fusion proteins (10C-10C, tfIII-10C, 10C-tfIII and rnc-10C). In summary, the testing of various fusion proteins produced interesting and unexpected results. We decided to focus on further characterization of the most promising fusion enzymes.

### Time course measurements to distinguish ligase activities of best performing 10C fusion enzymes

**Figure 3.**
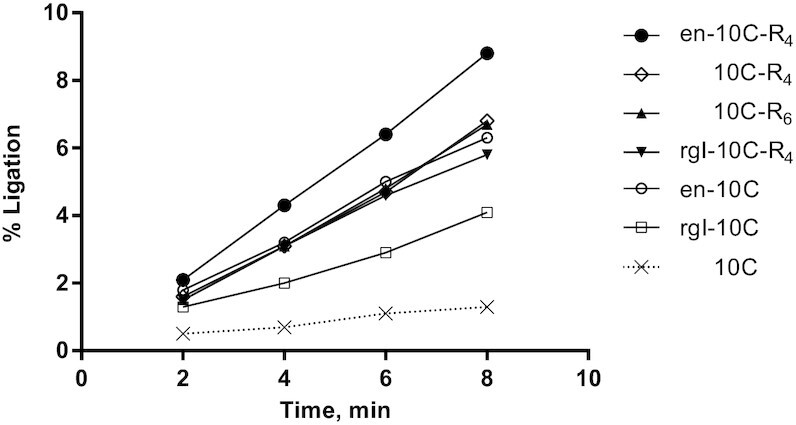
Time course of ligation reaction for promising ligase 10C fusions identified in initial single time point screening (Figure 2). Observed rate constant of ligase 10C alone was determined for comparison (dotted line). The best performing individual N- and C-terminal binding domains were combined and assayed as ligase 10C fusions en-10C-R_4_ and rgI-10C-R_4_. Assays contained 1 μM protein, 10 μM PPP-substrate, 15 μM DNA splint (DNA splint #2) and 20 μM OH-substrate (RNA-OH #2).

The best performing ligase 10C fusions zk-10C, 10C-hzk, en-10C, rgl-10C, rgII-10C, AR_4_-10C, 10C-R_4_, AR_6_-10C and 10C-R_6_ generated ∼40% or more ligation product in the screen described above (Figure 2). While the single time point screen identified enzymes with greater overall activity, a more detailed analysis was used to further distinguish their activities. For this purpose, four of the best performing N- and C-terminal fusions (en, rgl, R_4_, R_6_) were analyzed using a more detailed time course experiment. This assay was performed under modified conditions and with a series of shorter ligation times to compare the initial rate of the reaction at a substrate conversion of less than 10%. The time course assay confirmed the results of the initial activity screen for all fusions taken forward. The observed rate constants (*k*_obs_) for all the tested fusion proteins were 4- to 6-fold higher than the rate for ligase 10C (*k*_obs_ = 0.84 h^−1^) under the same conditions (Figure 3). The fusions enzymes with the highest initial activity were 10C-R_4_ and 10C-R_6_, both catalyzing the ligation reaction with a *k*_obs_ of ∼5.2 h^−1^. Whereas, the fusion protein en-10C had a slightly slower rate of ∼4.6 h^−1^. Additionally, we also constructed three-domain fusion proteins by combining ligase 10C alongside some of the best performing N- and C- terminal binding domains with the hope of increasing activity further (Table 1). The fusion protein rgI-10C-R_4_, showed no added improvement in ligation activity. Instead, the rate of ligation was slightly lower for rgI-10C-R_4_ (*k*_obs_ of ∼4.3 h^−1^) compared to 10C-R_4_ alone (*k*_obs_ of ∼5.2 h^−1^) (Figure 3). In contrast, the combination of same C-terminal R_4_ domain with the N-terminal binding domain, en yielded the fusion enzyme en-10C-R_4_ with an activity substantially higher (*k*_obs_ of ∼6.7 h^−1^) than that of the ligase 10C, or each of the binding domains fused separately (*k*_obs_ of ∼5.2 h^−1^). In this still relatively crude assay, the fusion protein en-10C-R_4_ ligated 8-fold faster than ligase 10C without any binding domains fused.

### Comparison of most active fusion enzymes to ligase 10C

**Figure 4.**
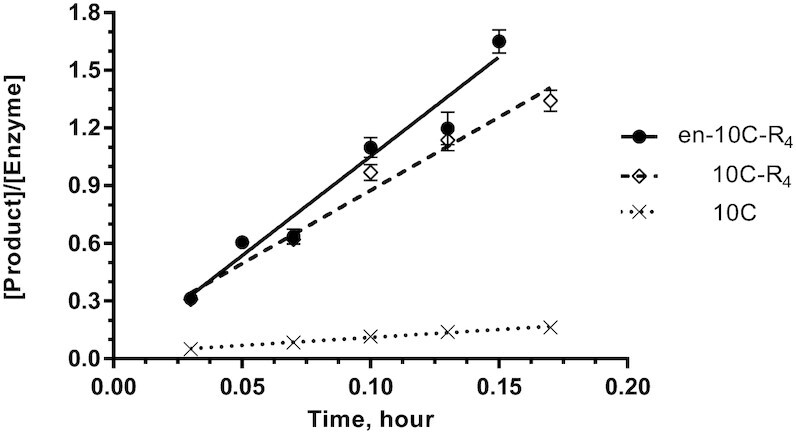
Time course of ligation reaction for the most active fusion enzymes. Observed rate constants were determined for ligase en-10C-R_4_, ligase 10C-R_4_ and the original ligase 10C without terminal substrate-binding domains. Assays contained 10 μM PPP-substrate, 15 μM DNA splint (DNA splint #2), 20 μM OH-substrate (RNA-OH #2) and either 5 μM ligase 10C, or 0.5 μM ligase 10C-R_4_ or ligase en-10C-R_4_. The error bars represent the standard error of the mean from two biological replicates with at least three technical replicates for each biological replicate.

The activity of the two most active fusion enzymes (en-10C-R_4_ and 10C-R_4_) identified in the time course screen above (Figure 3) were analyzed in more detail and compared to ligase 10C. Michaelis-Menten kinetics was not attainable for ligase 10C due to the sensitivity limitations of our gel shift assay as a result of the low substrate affinity of this enzyme. Therefore, to directly compare the ligation activity of ligase 10C to that of the fusion proteins 10C-R_4_ and en-10C-R_4_, we determined the *k*_obs_ for each enzyme variant. Ligation yield was measured over at least five time points within the linear range of substrate conversion for each enzyme (Figure 4). The more thorough *k*_obs_ measurements in Figure 4 showed reproducible reaction rates for all three fusion enzymes; ligase en-10C-R_4_ had a *k*_obs_ of 10.3 ± 0.6 h^−1^ whereas ligase 10C-R_4_ had a *k*_obs_ of 7.6 ± 0.4 h^−1^, corresponding to a 12- and 9-fold improvement in rate compared to ligase 10C with a *k*_obs_ 0.83 ± 0.04 h^−1^.

### Michaelis-Menten kinetics for ligase en-10C-R_4_

**Figure 5.**
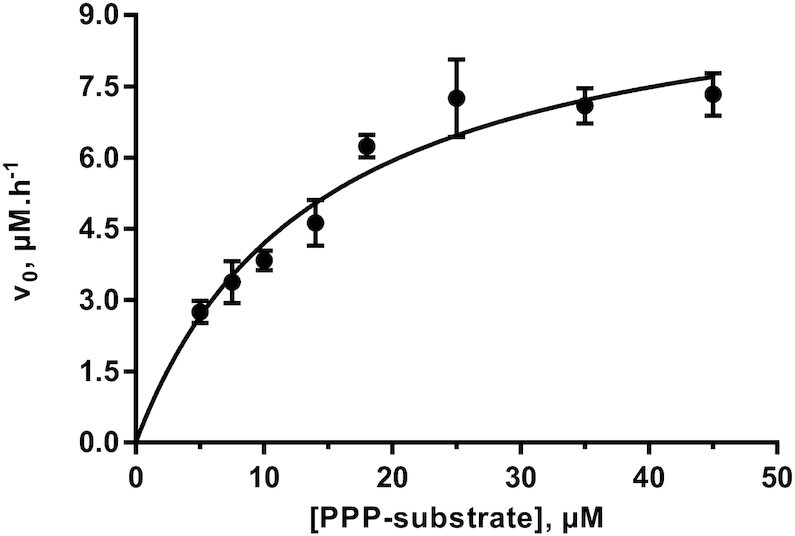
Initial reaction rate of reaction (*v*_0_) versus substrate concentration for ligase en-10C-R_4_. Assays contained 0.5 μM of enzyme together with PPP-substrate, DNA splint (DNA splint #2) and OH-substrate (RNA-OH #2) at a final molar ratio of 1:1.5:2. The error bars represent the standard error of the mean from two biological replicates with three technical replicates for each biological replicate. The data were fitted to the Michaelis–Menten kinetics model.

The combined fusion of the en and R_4_ domains improved the *k*_obs_ of the original ligase 10C by 12-fold. This improvement in activity enabled us to determine whether this completely *in vitro* evolved ligase en-10C-R_4_ reaction followed Michaelis-Menten catalysis and if so, how its catalytic efficiency compares to natural enzymes. The initial reaction rates at increasing substrate concentrations were fitted to the Michaelis-Menten model (Figure 5) and yielded a *k*_cat_ of 20 ± 1.6 h^−1^, a *K*_M_ of 14 ± 2.8 μM, and a *k*_cat_/*K*_M_ of 397 ± 159 M^−1^s^−1^.

### Nucleic acid binding affinity measurement by fluorescence anisotropy

**Figure 6.**
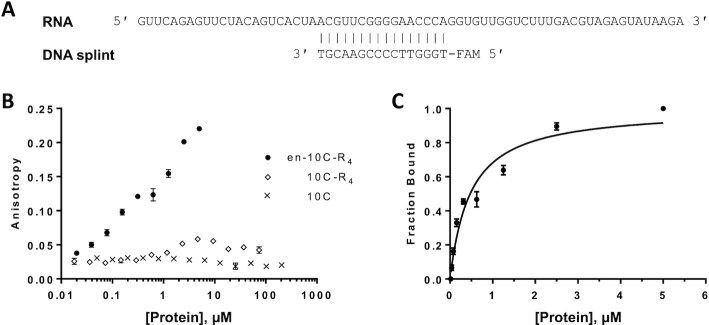
Binding affinity of ligase variants investigated by fluorescence anisotropy (FA). (**A**) RNA and complementary fluorescein-labeled DNA oligonucleotide (RNA/DNA duplex) used in FA assay. (**B**) Comparison of binding affinities of ligase 10C and its domain fusions en-10C-R_4_ and 10C-R_4_ to the RNA/DNA duplex. Two-fold dilution series of the enzymes were prepared in ligation buffer with 100 μg/ml bovine serum albumin and 30 nM of the annealed RNA/DNA duplex in 1:1 molar ratio. The error bars represent the standard error of the mean from two biological replicates with three technical replicates for each biological replicate. (**C**) Saturation binding curve of ligase en-10C-R_4_ calculated from FA data. The saturation curve was fitted to the direct binding model.

To verify that the 12-fold increase in activity of ligase en-10C-R_4_ is a result of the increased nucleic acid binding affinity caused by the two terminally-fused binding domains, the binding of en-10C-R_4_, 10C-R_4_ and ligase 10C was measured by fluorescence anisotropy (FA) (Figure 6). As the substrates themselves would react under the FA assay conditions, we used an RNA/DNA duplex that resembled the ligation product as a proxy for the substrate. The RNA in that duplex was identical to the ligated product, and the complementary DNA splint was identical to the DNA splint used in the ligation reactions except for a terminal fluorophore label to facilitate FA (Figure 6A). The dissociation constant (*K*_D_) of the most enzymatically active fusion enzyme en-10C-R_4_ for the RNA/DNA duplex was calculated from the FA curve as 460 ± 33 nM (Figure 6C). By comparison, ligase 10C without additional binding domains showed no increase in FA in the concentration range tested, thereby preventing a determination of its binding affinity (Figure 6B). Ligase 10C-R_4_ displayed a small increase in FA upon increasing protein concentration suggesting a weak binding affinity towards the RNA/DNA duplex; however, its binding curve could not be properly fitted to conventional binding models. These results indicated that the binding affinity of ligase 10C to the RNA/DNA duplex was much lower than that of both en-10C-R_4_ and 10C-R_4_.

## DISCUSSION

Natural proteins have been optimized by billions of years of natural evolution. Protein engineers, inspired by natural evolution, have carried out directed laboratory evolution to generate novel artificial enzymes. The artificial ligase 10C was created in the laboratory from a randomized protein library using a selection for product formation, but not for substrate binding (6,24,25,28). Consequently, ligase 10C was not optimized for substrate binding and likely possessed only weak affinity towards its substrate. We reasoned that a fusion of the artificial enzyme to a separate substrate-binding domain would increase the affinity of ligase 10C towards its substrates, thereby increasing its catalytic efficiency. We engineered and tested several variants of ligase 10C by fusing the protein to a variety of domains known to bind to nucleic acids in a sequence-independent manner such as RNA-binding domains, DNA-binding domains and arginine-rich peptides. The combination of the best performing binding domains yielded the fusion protein en-10C-R_4_, which outperformed the single domain enzyme, ligase 10C, by a 12-fold increase in ligation activity. These results demonstrate that the general principle of improving protein functionality through domain fusion, which has been observed in nature innumerous times, can be applied to improve the activity of artificial enzymes as well.

### Different substrate-binding domains exerted varied effects on catalytic activity

The chosen binding domains were fused to either, or both, the N- or C-terminus of ligase 10C. The binding domains effected the activity of ligase 10C differently. In general, improvements in ligation activity seemed to correlate with the fusion of smaller binding domains. The natural and synthetic arginine-rich peptide motifs improved ligation activity the most consistently, with the C-terminally fused R_6_ peptide displaying the greatest increase in ligation yield. Overall, the activity of fusions with arginine-rich peptides outperformed the activity of other binding domains tested, except for the N-terminally fused DNA-binding domain en, the RNA binding domain zk, and the C-terminally fused RNA binding domain hzk.

The many examples of multi-domain proteins in nature (2) and examples of successfully engineered RNA-binding proteins (10,11,45) demonstrate the interchangeable nature of binding domains. Yet, an RNA-binding domain we tested as a fusion with the artificial ligase 10C did not improve its activity, but instead decreased activity (rnc). This relatively large binding domain may have altered the conformation of the ligase to a less active from. Alternatively, it is possible that the fusion of this relatively large RNA-binding domain to the smaller ligase 10C led to steric clashes and hindered the accessibility of the enzyme's active site. The substrate may still bind to the fused binding domain but not in the correct position for ligation to occur efficiently. However, we also found an example that did not follow this apparent size-dependent trend: the similarly large en domain was successful as an N-terminal fusion. It appears that, regardless of size, the structure of a given substrate-binding domain might determine whether it is compatible with the active site of the ligase.

### Combining binding domains yielded most efficient enzyme

The activity of the ligase was further improved by combining two binding domains from the better-performing N- and C-terminal fusion enzymes. The simultaneous fusion of both the R_4_ and en domains to ligase 10C yielded an additive effect on activity. The *k*_obs_ of the resulting three-domain enzyme en-10C-R_4_ had increased by 12-fold compared to the original ligase 10C, and 1.4-fold compared to the most improved single domain fusion 10C-R_4_. The improvement in ligation activity for en-10C-R_4_ also enabled us to determine a full Michaelis-Menten kinetics analysis. In contrast, it was not possible to determine the Michaelis-Menten kinetics parameters for ligase 10C due to the high concentration of substrate that this would have required.

The 12-fold increase in activity for our artificial enzyme favorably compared to a previous study that increased the activity of the natural enzyme T4 DNA ligase for blunt end ligation by 7-fold through fusion to DNA-binding domains (23). While the reaction mechanisms of these two enzymes are likely different as T4 DNA ligase requires ATP as cofactor, the same approach of domain fusion successfully increased ligation activity of both the naturally evolved and the artificial enzyme described here.

The catalytic efficiency (*k*_cat_/*K*_M_) of the majority of enzymes in nature ranges between 10^3^ and 10^6^ M^−1^ s^−1^ (46). The improvement of our artificial ligase resulted in en-10C-R_4_ with a *k*_cat_/*K*_M_ of 397 ± 159 M^−1^s^−1^, which is just below this range by only 3-fold. This increase in activity will further facilitate the use of the artificial ligase in applications like RNA sequencing and the detection of 5′-triphosphorylated RNA (26).

### Increased nucleic acid binding affinity correlated with improved ligase activity

We observed a gradual increase in binding to the RNA/DNA substrate proxy when comparing ligase 10C, 10C-R_4_ and en-10C-R_4_: no detectable binding for ligase 10C, low binding for 10C-R_4_, and substantially increased binding for en-10C-R_4_ (Figure 6). The catalytic activity of these three variants increased in the same order (Figure 4). This correlation thereby confirmed the underlying assumption made at the start of this project. While we verified this general correlation between increasing affinity and increasing catalytic activity, the correlation is not linear. For example, the fusion of just the arginine domain R_4_ to ligase 10C already increased the catalytic activity by 9-fold, which is close to the 12-fold increase for en-10C-R_4_. However, the binding affinity of 10C-R_4_ is substantially lower than that of en-10C-R_4_ (Figure 6B). For the reasons stated above, the binding measurements had to be carried out using an RNA/DNA duplex of the ligated product. Both the substrate and product are polyanionic nucleic acids and therefore similar with respect to their binding to positively charged arginine residues. Nonetheless, the binding measurements are a reflection of the affinity for the product rather than the substrates. The ligase en-10C-R_4_ had a dissociation constant (*K*_D_) of 460 ± 33 nM for the product while its Michaelis–Menten constant (*K*_M_) was 14 ± 2.8 μM. It is possible that increasing the catalytic activity through increasing the substrate affinity has diminishing returns because of eventual inhibition by substrate and/or product. The *k*_obs_ for en-10C-R_4_ at 75 μM of PPP substrate was 30% lower than the *k*_cat_ determined in the Michaelis–Menten assay indicating substrate inhibition at high substrate concentrations. This *k*_obs_ data point was excluded from the Michaelis–Menten kinetics fitting. The substrate inhibition could be caused by unspecific interaction between the enzyme and the substrate. The occurrence of substrate inhibition also indicates that the approach of increasing enzyme activity through increasing the substrate affinity has some limitation.

### Engrailed homeodomain interaction with RNA and its primordial relevance

The engrailed homeodomain (en) has previously been characterized mostly as a DNA-binding domain (33,40,41). Therefore, the improved performance of the en-10C fusion enzyme was somewhat unexpected and provides the first direct evidence that the helix-turn-helix (HTH) homeodomain can increase the activity of an RNA-acting enzyme. The dissociation constant for en-10C-R_4_ of 460 ± 33 nM is similar to the published *K*_D_ value for the en domain binding to a DNA/DNA duplex (47).

The HTH fold is highly evolutionary conserved and as such the fold has been suggested to be of ancient origin (48). Modern protein folds are thought to have emerged from the joining of short length peptides that were present as cofactors in the RNA world (48). An extensive analysis of evolutionary conserved folds identified HTH as one of the most likely abundant primordial peptide motifs (48). Furthermore, a separate study has found that the dsRNA-binding surface of the ribonucleoprotein L11 resembles the HTH homeodomain (42). Ordinarily, the HTH binds to the DNA major groove of a B-form helix, which is much larger than the major groove an A-form helix formed by dsRNA (or an RNA/DNA heteroduplex). NMR studies suggested that the binding of L11 to dsRNA might be facilitated through a distortion of the A-form helix in this complex resulting in more of a B-form conformation (43). Further studies along similar lines would be required to identify the mechanism of how the en domain interacts with the RNA substrate of the primordial model enzyme ligase 10C. Ribonucleoproteins like L11 are commonly thought to have existed in the ancient RNA world and enabled the transition from that RNA world to the DNA/protein-dominated contemporary biology.

### Coevolution of domains in modern and primordial proteins

The artificial ligase 10C used as the starting point for this study was generated in a test tube by *in vitro* selection. In contrast to the evolution of natural proteins over billions of years, the ligase was only subjected to three rounds of directed evolution (6,25) and has therefore been referred to as a primordial-like protein (27). Ligase 10C can be used as a model protein to investigate how domain fusion affects the activity of a primordial protein, and to shine light on how the evolution of multi-domain proteins might have started.

Domain fusion is a common strategy in natural protein evolution. However, the ability for protein families to swap or incorporate different domains through recombination has limitations. Analysis of protein superfamilies has previously shown that the recombination of domains is conditional on certain folds (2). This finding suggested that certain combinations of domains are evolutionary conserved and not all domains can interchange with all other domains. Other research highlighted that high conservation in multidomain proteins is focused at the domain-binding interface and domain linker regions, therefore lending further support to this concept (49). The positive and negative effects of different fusion domains on the activity of the artificial enzyme described in our current study extends this concept to primordial-like proteins. All the short natural and synthetic arginine-rich peptide motifs (<3 kDa) that we tested increased the ligation activity of ligase 10C, in contrast to only one of the binding domains larger than >7 kDa (en). This finding suggests that the addition of simple arginine-rich sequences or domains to a primordial-like enzyme can be as powerful for improving activity as swapping in a more complex protein domain (en). As simple sequences have a higher probability to emerge by chance, this path to improve function is especially useful during the early stages of protein evolution. These short natural RGG/RG box motifs have been described to be intrinsically disordered and highly flexible (50). The synthetic arginine-rich peptides are expected to behave similarly. The relatively small ligase 10C appears to benefit from a fusion to mostly small, flexible and unstructured substrate-binding domains. These features of the arginine-rich peptides are reminiscent of how one might imagine simple primordial peptides. The primordial-like binding motifs having a greater impact on improving the catalytic activity of our primordial-like protein further highlights the importance of domains to co-evolve.

### Conclusions

Inspired by nature's success in producing specialized multi-domain proteins, we enhanced the activity of the artificial ligase 10C by improving its substrate binding affinity. We thereby demonstrated for the first time that an artificial, primordial-like enzyme can be optimized through domain fusion. The catalytic efficiency of the resulting three-domain fusion enzyme is close to the range of catalytic efficiencies of average natural enzymes. The primordial-like, artificial protein was shown to form improved fusion proteins with some - but not all - substrate binding domains, which is similar to what has been observed for naturally evolved proteins. In summary, these results suggest that domain fusion likely has played an important role already in early protein evolution.

## DATA AVAILABILITY

Data are available as supplementary data. The GenBank accession number for ligase en-10C-R_4_ is OM304634.

## Supplementary Material

gkac858_Supplemental_File
