# Supplementary material for: Nature-inspired engineering of an artificial ligase enzyme by domain fusion
Source: Nucleic Acids Res. 2022 Oct 16;50(19):11175–85. doi: 10.1093/nar/gkac858 (PMC9638898; doi:10.1093/nar/gkac858)
Supplement: gkac858_Supplemental_File [file gkac858_supplemental_file.docx]

SUPPLEMENTARY INFORMATION

**Nature-inspired engineering of an artificial RNA ligase enzyme by domain fusion**

Cher Ling Tong^1,2,*^, Nisha Kanwar^1,2,*,#^, Dana J. Morrone^1,2,*^, & Burckhard Seelig^1,2,#^

^1^ Department of Biochemistry, Molecular Biology and Biophysics, University of Minnesota, Minneapolis, Minnesota, 55455, USA. ^2^ BioTechnology Institute, University of Minnesota, St. Paul, Minnesota, 55108, USA.

* These three authors contributed equally.

^#^ To whom correspondence should be addressed. Email: [seelig@umn.edu](mailto:seelig@umn.edu), [nisha.kanwaruk@gmail.com](mailto:nisha.kanwaruk@gmail.com)

**Contents:**

Supplementary Table S1 – S2

Supplementary Figures S1 – S2

**Supplementary Table S1.** All oligonucleotides / primers

**Supplementary Table S2.** Protein sequences of ligase 10C and fusion proteins comprising ligase 10C and different substrate-binding domains. The binding domain sequences are shown in bold and the flexible linker sequences are shown in green.

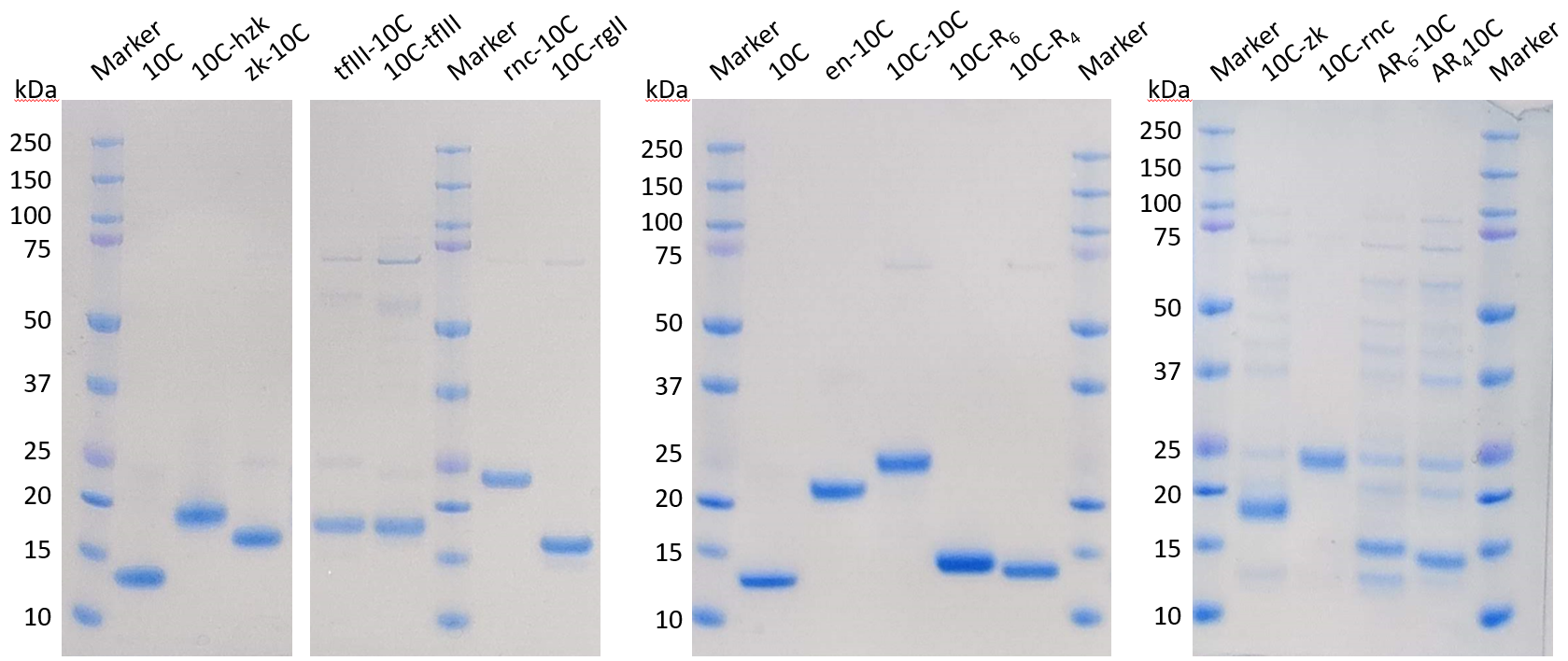


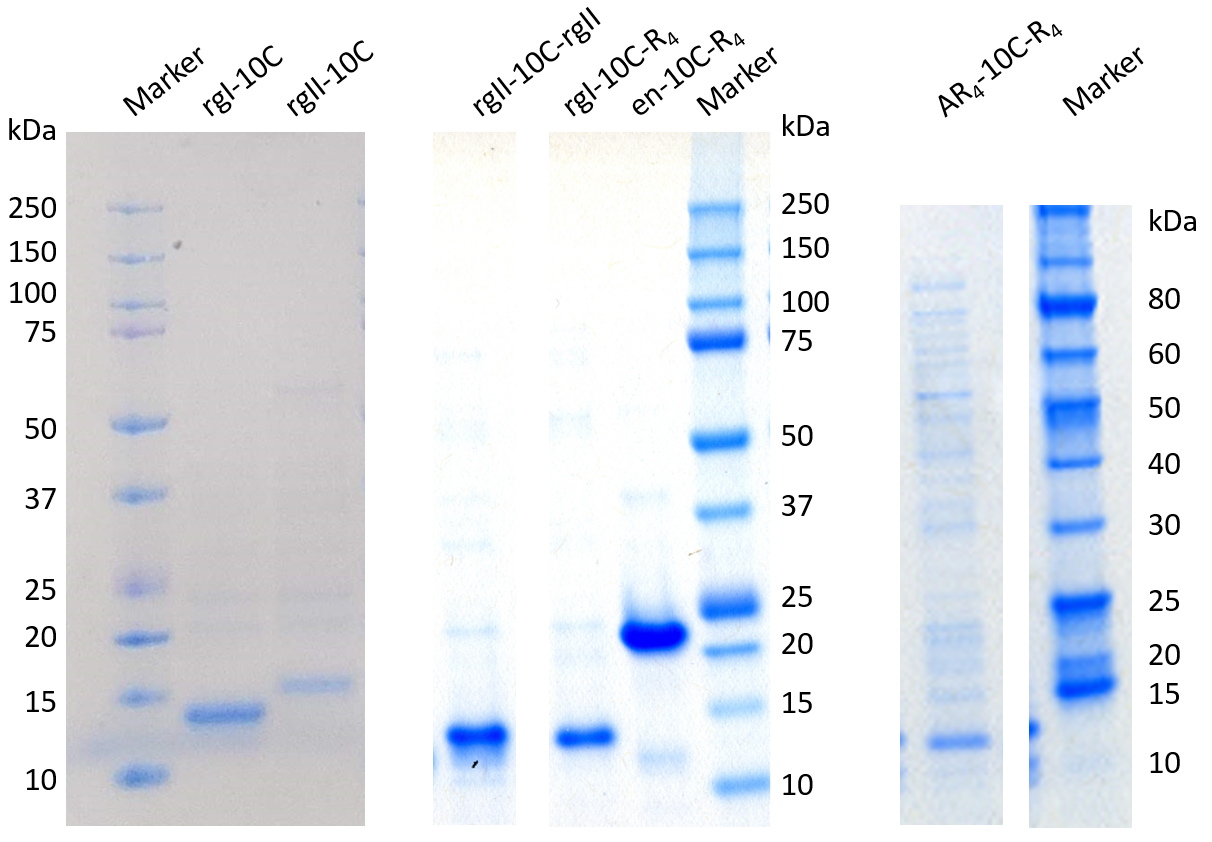


**Supplementary Figure S1.** SDS-PAGE gels of ligase 10C fusion proteins used in this study. The proteins were purified by Ni-NTA chromatography, dialyzed and concentrated.


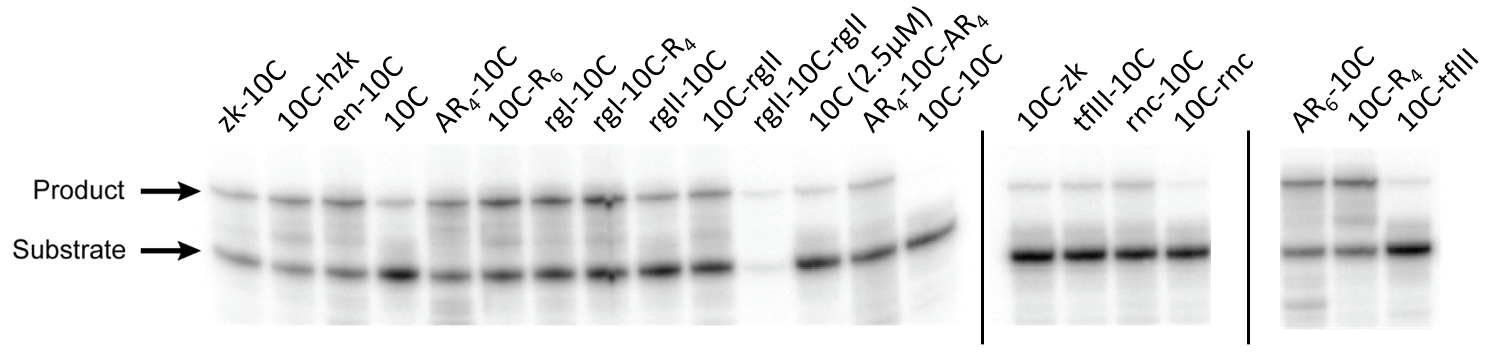


**Supplementary Figure S2.** Initial screen for ligation activity of all soluble fusion proteins carried out as single time point assay. Radiolabeled substrate and product RNA were separated on a Urea PAGE gel after a 1 hour ligation reaction. All assays were done with 5 µM of protein unless mentioned otherwise in the figure. A representative gel is shown in this figure.
